# Deep Learning Reconstruction for ^129^Xe Diffusion‐Weighted MRI Enables Use of Natural Abundant Xenon and Improved Image Acceleration

**DOI:** 10.1002/mrm.70194

**Published:** 2025-11-20

**Authors:** Rajagopalan Sundaresan, Guilhem J. Collier, Neil J. Stewart, Sudhanya Chatterjee, Ramesh Venkatesan, Jan Wolber, Jim M. Wild

**Affiliations:** ^1^ POLARIS, Division of Clinical Medicine, School of Medicine & Population Health, Faculty of Health The University of Sheffield Sheffield UK; ^2^ GE HealthCare Bengaluru India; ^3^ Insigneo Institute The University of Sheffield Sheffield UK; ^4^ GE HealthCare Chalfont St Giles UK

## Abstract

**Purpose:**

(i) To assess whether ^129^Xe apparent diffusion coefficient (ADC) and diffusive length scale (Lm_D_) metrics are quantitatively preserved with deep learning (DL) accelerated acquisition and reconstruction and (ii) to evaluate the feasibility of ^129^Xe diffusion weighted imaging with natural‐abundance xenon at increased acceleration factors.

**Methods:**

Twenty three‐dimensional compressed sensing (CS) accelerated ^129^Xe DW MRI datasets were gathered from a cohort of patients with asthma, chronic obstructive pulmonary disease (COPD) and idiopathic pulmonary fibrosis (IPF). Images were retrospectively reconstructed with DL based CS, denoising and de‐ringing reconstruction, and compared to conventional CS. ADC and diffusive length scales (Lm_D_) were assessed and compared between conventional CS and DL reconstructions. Prospectively acquired DL reconstruction was then assessed in three healthy volunteers who underwent ^129^Xe DW MRI with both natural‐abundance and enriched xenon mixes.

**Results:**

DL reconstruction qualitatively improved the sharpness, SNR and image quality of ^129^Xe DW images. In the retrospective study, DL reconstruction produced a slight bias in ADC (5.4%) and Lm_D_ (0.8%) values when compared with conventional CS reconstruction. In the prospective study, DL reconstruction significantly improved the SNR of natural‐abundance xenon images and produced ADC and Lm_D_ values comparable to those achieved with 129‐enriched xenon.

**Conclusion:**

DL‐based CS, denoising and de‐ringing significantly improves SNR and image sharpness in 3D ^129^Xe diffusion‐weighted MRI while exhibiting a slight bias in ADC and Lm_D_. This approach enables the use of natural‐abundance xenon and higher acceleration factors, offering substantial cost reduction and improved clinical feasibility for hyperpolarized ^129^Xe lung morphometry.

## 1 Introduction

Hyperpolarized ^129^Xe magnetic resonance imaging (MRI) is a valuable modality to assess lung function and structure [1, 2, 3, 4]. Diffusion weighted (DW) MRI with hyperpolarized ^129^Xe enables quantitative assessment of lung microstructure through metrics such as the apparent diffusion coefficient (ADC) and morphometry measurements generated from diffusion models [5, 6, 7, 8, 9]. The sensitivity of ^129^Xe DW MRI in probing tissue microstructure has been well established in patients with chronic obstructive pulmonary disease (COPD) and emphysema [1, 2]. Hyperpolarized gas diffusion biomarkers also agree well with histologically derived measurement parameters [2, 6]. However, ^129^Xe DW‐MRI faces significant SNR challenges due to the low gyromagnetic ratio and diffusivity, which necessitates longer diffusion gradient times, and in turn, sequence echo time (TE) and repetition times (TR) [9] that together extend the acquisition time needed for volumetric coverage of the lungs. Several research groups have worked on multi *b* value ^129^Xe DW MRI [6, 9, 10] with the different *b* value interleaves either acquired without whole lung coverage or across multiple breath‐holds with long scan times. 3D spoiled gradient echo (SPGR) acquisitions [6, 10] with compressed sensing (CS) acceleration were proposed to enable whole lung coverage, but the breath‐holds for such scans are longer (> 4 s per interleave) [6] and have the same SNR limitations. Though CS techniques have been developed to accelerate hyperpolarized gas MRI acquisitions [6, 10, 11, 12] the resulting SNR penalty further constrains the achievable acceleration factors, especially for diffusion‐weighted acquisitions where SNR is intrinsically lower. Current clinical scans with CS acceleration factor (AF) of four require breath‐holds of approximately 17 s when implemented as per [6]. This duration can be challenging for patients with respiratory limitations and exceeds the recommended breath‐hold duration of less than 15 s established by the ^129^Xe MRI clinical trials consortium protocols [13].

To address SNR limitations, most ^129^Xe diffusion imaging studies utilize enriched (EN) xenon mixes (> 85% ^129^Xe) compared to natural‐abundance (NA) xenon (26% ^129^Xe) [8] as enriched xenon provides an approximately threefold SNR benefit. However, natural abundance xenon has been historically an order of magnitude cheaper than enriched xenon [13] and with improvements in polarization technology [14], it can be a viable option for ^129^Xe ventilation imaging [15]. NA ^129^Xe ventilation imaging has recently been shown to be improved with a deep learning denoising and de‐ringing reconstruction pipeline [16] and CS reconstruction has enabled robust NA ^129^Xe gas exchange imaging [12] but the feasibility of diffusion weighted NA ^129^Xe imaging, (where quantitative accuracy is crucial), has yet to be demonstrated. Importantly, DL reconstruction has the potential to enable higher acceleration factors which could reduce breath‐hold time within the consortium guidelines of 15 s while maintaining or improving image quality.

Some research groups have focussed on use of deep learning only for ^129^Xe diffusion CS reconstruction [17] and explored algorithmic denoising techniques like HOSVD [18] to improve diffusion image quality, but there has been no reported work on an integrated reconstruction approach that employs DL‐based CS, denoising and de‐ringing methods together. While the present manuscript was under review, a publication comparing supervised and unsupervised deep learning strategies for denoising ^129^Xe images was published [19]. While the approach used in that work addressed only denoising of ^129^Xe images, our deep learning approach involves an integrated reconstruction as follows.

In this work, we evaluate the feasibility of applying deep learning‐based CS, denoising and de‐ringing to ^129^Xe diffusion‐weighted MRI. Mean ADC and Lm_D_ values are compared between conventional CS reconstruction and DL reconstruction in a retrospective patient cohort. We demonstrate that these combinations of techniques enable: (1) the use of natural‐abundance xenon instead of enriched xenon; and (2) increased acceleration factors while maintaining quantitative diffusion metrics.

## 2 Methods

### 2.1 Retrospective Study Design

A retrospective analysis of 20 ^129^Xe diffusion datasets was conducted from the Advanced Diagnostic Profiling (ADPro) study [20]. The patients were scanned under a National Research Ethics Committee approved protocol (16/EM/0439) and had a diagnosis of asthma and/or COPD. The datasets were selected manually to cover a range of Lm_D_ values from a database of > 100 examinations and were retrospectively reprocessed using the deep learning reconstruction pipeline. To demonstrate applicability to other lung disorders, we also retrospectively analyzed a dataset from a patient with idiopathic pulmonary fibrosis (IPF) [21].

### 2.2 Prospective Study Design

A prospective study then followed to assess the feasibility of using natural abundance xenon and CS acceleration factor of 5. Three healthy volunteers (age: 29, 35, 36 years; sex: 3 M) were scanned with a 50:50 mix of natural abundance xenon (550 mL) and N_2_ (450 mL). Hyperpolarized ^129^Xe doses were produced to a polarization level of 30% using an in‐house regulatory approved spin‐exchange optical pumping polarizer [14]. All volunteers were also scanned with the usual dose of 129‐enriched Xenon. The volunteers were scanned under ethics UOS052024. Four datasets were acquired from the three volunteers with both enriched and natural abundance xenon: three datasets using conventional acceleration factor of 4 and one dataset using acceleration factor of 5. The CS masks used with acceleration factors of 4 and 5 are shown in Figure S3.

### 2.3 MRI Acquisition

Images were acquired on 1.5T GE Healthcare scanners (HDx for the retrospective study and SIGNA Artist for the prospective study) with a flexible single channel transmit‐receive vest coil (Clinical MR Solutions). For the retrospective study, only 129‐enriched xenon was used (˜86% enrichment) and for the prospective study, both 129‐enriched xenon and natural abundance (26% ^129^Xe) xenon were used. In all cases, the total gas volume delivered was determined based on patient height as previously described in [22], with volumes typically ≤ 1 L. The gas was inhaled from functional residual capacity with subjects maintaining a breath‐hold during image acquisition.

Volumetric multiple *b* value diffusion‐weighted ^129^Xe MRI was acquired using a 3D spoiled gradient echo sequence with the following parameters: Average FOV = 40 × 32.5 × 27 cm^3^; matrix = 64 × 52 × 18 (effective voxel size = 6.25 × 6.25 × 15 mm^3^), elliptical‐centric phase encoding, four diffusion‐weighted interleaves (*b* = 0, 12, 20, and 30 s/cm^2^), CS acceleration factor (AF) = 4, TE/TR = 11.7/15.0 ms, diffusion time (Δ) = 8.5 ms, and flip angle = 2.7°. This protocol was adapted from Chan et al. [6].

### 2.4 Image Reconstruction

Raw *k*‐space data from each acquisition were reconstructed using:

1. Conventional CS reconstruction [6, 10].
2. Deep learning‐based (DL) reconstruction pipeline.

The integrated DL reconstruction pipeline combines a supervised DL network (adapted from [23] for single channel use) trained to fill missing *k*‐space points (hereafter referred to as DL CS) and, subsequently, AIR Recon DL [24] (GE Healthcare) for denoising and de‐ringing. AIR Recon DL utilizes a convolutional neural network (CNN) containing 4.4 million trainable parameters across approximately 10 000 kernels designed for MRI reconstruction. The key features of the CNN include: scale‐invariant design using Rectified Linear Unit (ReLU) activations without bias terms, enabling effective processing across the wide dynamic range encountered in low‐SNR ^129^Xe imaging, providing user‐tunable noise reduction and integrated truncation artifact suppression that converts ringing artifacts into improved edge sharpness. The network was trained using supervised learning with pairs of near‐perfect high‐resolution and lower‐resolution MR images with added truncation artifacts and noise, utilizing the ADAM optimizer to minimize mean squared error between CNN predictions and target images [24]. For the current study, we employed the commercially available implementation without modification or retraining specific to ^129^Xe imaging, applying maximum denoising (1.0) and de‐ringing (1.0) levels based on the low‐SNR characteristics of hyperpolarized gas diffusion imaging. Images were also generated after DL CS only, DL CS with only denoising (DL CS: DN) and DL CS with denoising and de‐ringing (DL CS: DN + DR) to isolate and characterize the effects of the individual processing steps.

### 2.5 Data Analysis

ADC maps from the *b* = 0 and *b* = 12 s/cm^2^ images were calculated. For multiple *b* value analysis, the stretched exponential model (SEM) was applied to the diffusion signal decay:

SbS0=e−(b*DDC)α

where *S*_0_ is the signal at *b* = 0, *S*_*b*_ is the signal at a non‐zero *b* value, DDC is the distributed diffusivity coefficient, and *α* is the heterogeneity index (ranging from 0 to 1). Unlike the standard mono‐exponential model, the SEM accounts for the heterogeneous microstructural environment within each voxel. From these parameters, we derived the mean diffusive length scale (Lm_D_) which represents the alveolar dimensions. The SEM analysis was done using custom software written in MATLAB.

For the retrospective study, Bland–Altman analysis was performed to assess the agreement between different reconstruction methods for both ADC and Lm_D_ maps. This analysis was crucial for determining whether the DL reconstruction introduced any systematic bias into the quantitative diffusion measurements. A simple flowchart showing the process of reconstruction and image analysis is included in Figure S1.

### 2.6 Synthetic Noise Addition Experiment

To evaluate the robustness of the DL reconstruction at different noise levels, we added complex Gaussian noise with standard deviations of: 2×, 3.3× (which represents the expected SNR drop when using natural abundance xenon) and 5× the original noise level, to one COPD dataset obtained with 129‐enriched xenon. The datasets with added noise were reconstructed using conventional CS and the DL reconstruction methods, and the resulting ADC and Lm_D_ maps were compared.

## 3 Results

### 3.1 Retrospective Study

**FIGURE 1:**
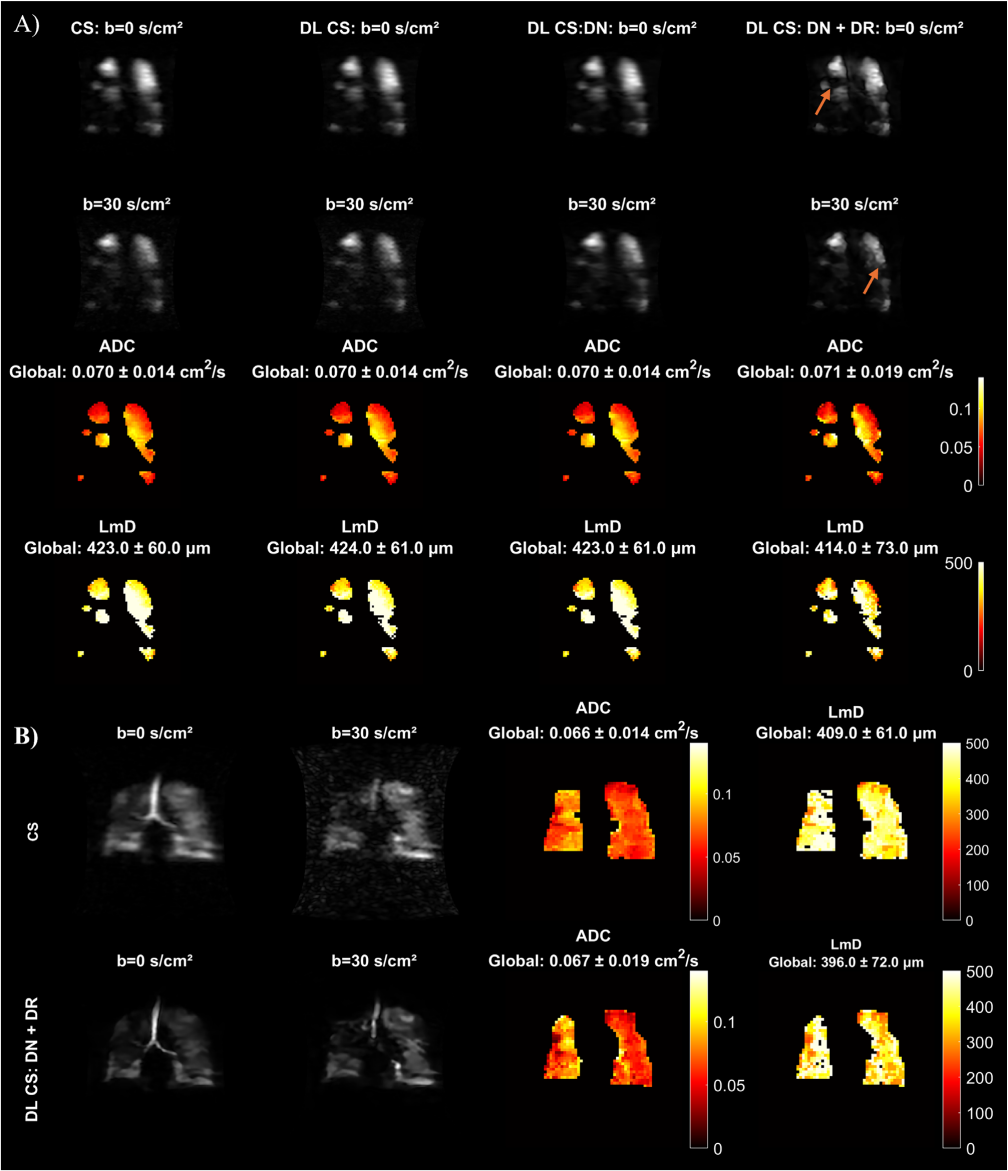
Retrospective study. (A) From left to right: Conventional CS reconstruction, deep learning CS reconstruction (DL CS), deep learning CS reconstruction with denoising (DL CS: DN), deep learning CS reconstruction with denoising and de‐ringing (DL CS: DN + DR) for *b* = 0 s/cm^2^ and *b* = 30 s/cm^2^ images. The corresponding ADC and Lm_D_ maps are shown for a COPD patient. The global ADC and Lm_D_ values are indicated for each reconstruction method. Arrows indicate enhanced image sharpness achieved with deep learning reconstruction. (B) Representative results from an Idiopathic pulmonary fibrosis (IPF) patient. Images for *b* = 0 s/cm^2^, *b* = 30 s/cm^2^, ADC and Lm_D_ maps are shown for conventional CS reconstruction and DL CS: DN + DR.

^129^Xe diffusion images from a COPD patient are shown in Figure 1A.

The figure presents images from conventional CS reconstruction, DL‐based CS reconstruction (DL CS), deep learning CS reconstruction with denoising (DL CS: DN), and deep learning reconstruction with denoising and de‐ringing (DL CS: DN + DR). The *b* = 0 and *b* = 30 images are shown along with the corresponding ADC and Lm_D_ maps. The DL CS: DN + DR reconstructed images demonstrate substantial visual quality enhancement with increased sharpness indicated by arrows. In addition to the enhanced image appearance with the DL reconstruction, the spatial distributions of quantitative diffusion metrics ADC and Lm_D_ were well preserved.

Figure 1B shows results from an IPF patient. The *b* = 0, *b* = 30 images are shown with the corresponding ADC and Lm_D_. Similar observations of improved image quality and sharpness with preservation of quantitative metrics were observed.

**FIGURE 2:**
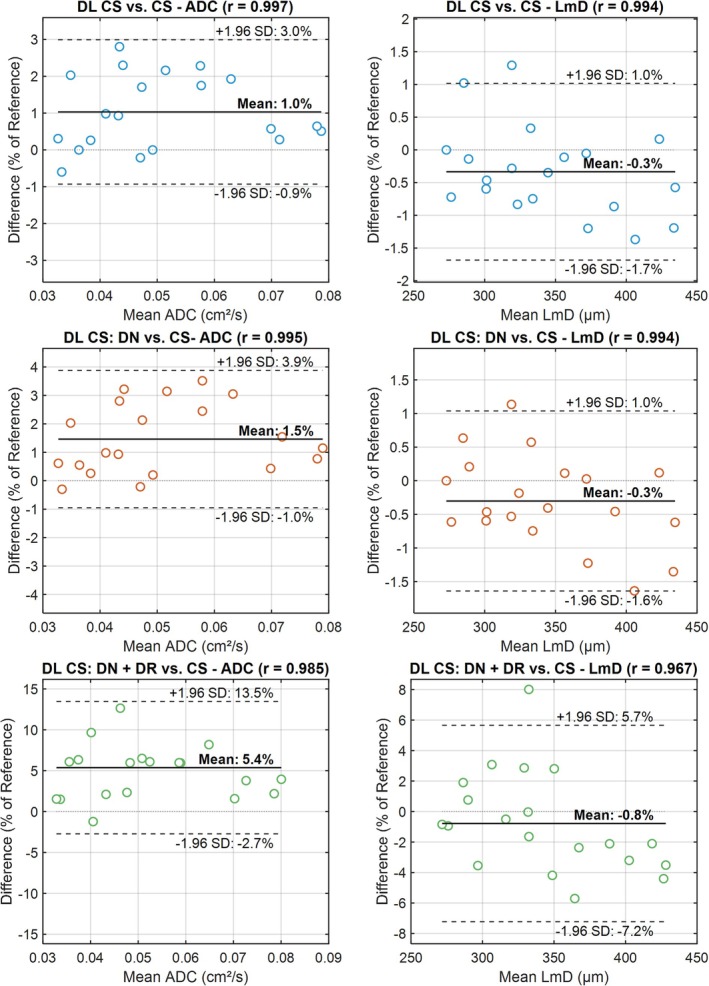
Retrospective study. Bland–Altman plots of the percentage differences in apparent diffusion coefficient (ADC) and mean diffusion length scale (Lm_D_) as a function of their mean values calculated from conventional CS reconstruction (CS), deep learning CS reconstruction (DL CS), deep learning CS reconstruction with denoising (DL CS: DN) and deep learning CS reconstruction with denoising and de‐ringing (DL CS: DN + DR) for *n* = 20 patients with asthma and/or chronic obstructive pulmonary disease. The correlation coefficients *r* between the measurements are shown along with the mean and limits of agreement in solid lines and dashed lines respectively.

Across the 20 datasets, the ADC and Lm_D_ values derived from the DL reconstruction pipelines showed good agreement with the conventional CS results, with a slightly increased bias for DL CS: DN + DR. To quantify the bias, Bland Altman analyses were performed. Figure 2 shows the Bland–Altman plots for DL CS, DL CS: DN, and DL CS: DN + DR.

**FIGURE 3:**
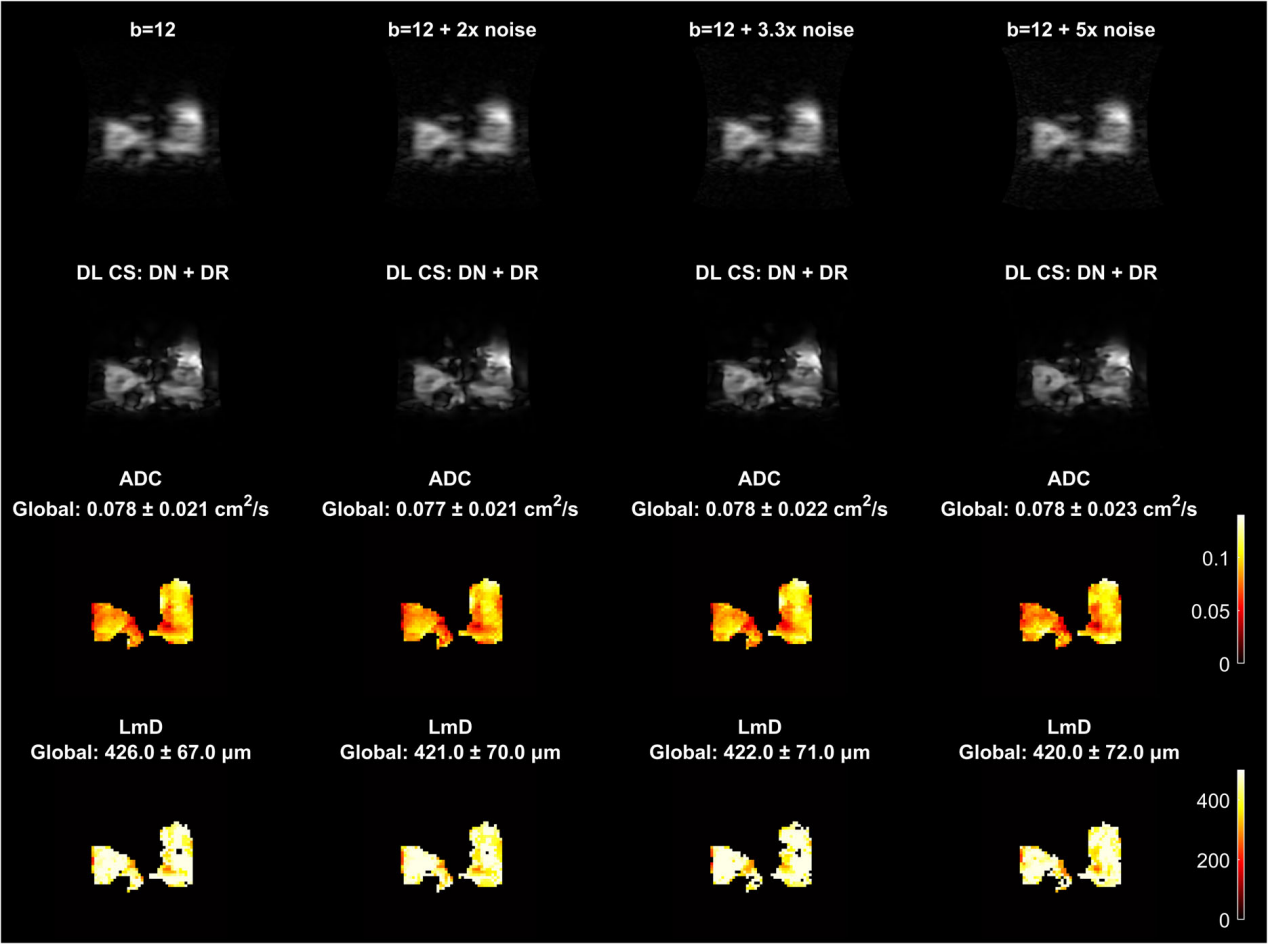
Retrospective study. Deep learning reconstruction with Gaussian noise added to acquired *k*‐space on a COPD dataset. The figure shows *b* = 12 s/cm^2^ images with 2×, 3.3×, and 5× noise added, and corresponding images with DL CS: DN + DR. ADC and Lm_D_ maps reconstructed with DL CS: DN + DR are also shown. Note that addition of 3.3× noise is chosen to mimic acquiring with natural abundant xenon.

Comparison of ADC values with DL CS: DN + DR and conventional CS reconstruction revealed a mean positive bias of 0.003 cm^2^/s (5.4%) which reduced to 0.0008 cm^2^/s (1.46%) when de‐ringing was removed. Similarly, the mean Lm_D_ difference between CS and DL CS: DN + DR showed a negative bias of 3.5 μm (0.78%) which reduced to 1.2 μm (0.3%) when de‐ringing was removed. The mean ADC and Lm_D_ differences between CS and DL CS were 1% and 0.33%, respectively which suggests that CS and DL CS produced nearly identical results. Figure 3 shows the results of the synthetic noise addition experiment on a COPD dataset.

Qualitatively, the reconstructed images look sharp even after addition of noise. The mean global ADC values obtained from DL reconstruction showed no differences at 2×, 3.3×, and 5× noise levels, respectively, compared to the DL reconstruction without adding noise. The mean global Lm_D_ showed negligible differences at 2×, 3.3×, and 5× noise levels with mild regional heterogeneity that became slightly less similar with increasing noise levels. To quantify the regional variations, we computed the mean absolute error (MAE) of the ADC and Lm_D_ maps at 2×, 3.3×, and 5× noise levels compared to the DL reconstruction without addition of noise. The MAE in ADC was 2.9e−4, 4.34e−4, and 5.54e−4 cm^2^/s while the MAE in Lm_D_ was 2.86, 4.19, and 5.06 μm at 2×, 3.3×, and 5× noise levels, respectively.

### 3.2 Prospective Study

Table 1 summarizes the global mean ADC (ADC_glob_) and Lm_D_ (Lm_Dglob_) values obtained from three healthy volunteers using a natural abundance Xe + N_2_ inhaled gas mixture.

**TABLE 1:** Global mean ADC (ADC_glob_) and Lm_D_ (Lm_Dglob_) from three healthy volunteers obtained with enriched and natural abundance Xe + N_2_ inhaled gas mixture.

Subject	1 (EN)	1 (NA)	2 (EN)	2 (NA)	3 (EN)	3 (NA)
ADC_glob_ (cm^2^/s) (DL CS)	0.030 ± 0.008	0.029 ± 0.009	0.042 ± 0.008	0.043 ± 0.015	0.033 ± 0.010	0.034 ± 0.008
ADC_glob_ (cm^2^/s) (DL CS: DN + DR)	0.030 ± 0.013	0.033 ± 0.014	0.042 ± 0.011	0.047 ± 0.019	0.036 ± 0.012	0.033 ± 0.013
Lm_Dglob_ (μm) (DL CS)	261 ± 62	255 ± 67	320 ± 54	318 ± 63	274 ± 60	281 ± 54
Lm_Dglob_ (μm) (DL CS: DN + DR)	257 ± 72	272 ± 79	318 ± 67	328 ± 76	289 ± 72	273 ± 71

The mean ADC and Lm_D_ values were preserved between enriched and natural abundance xenon with DL CS: DN + DR when compared to DL CS reconstruction. The *b* = 0 images, ADC and Lm_D_ maps are shown for one volunteer in Figure S2 where the use of DL CS: DN + DR shows slight visual variations in the ADC and Lm_D_ maps while it increased the SNR of natural abundant xenon to be closer to that of enriched xenon.

**FIGURE 4:**
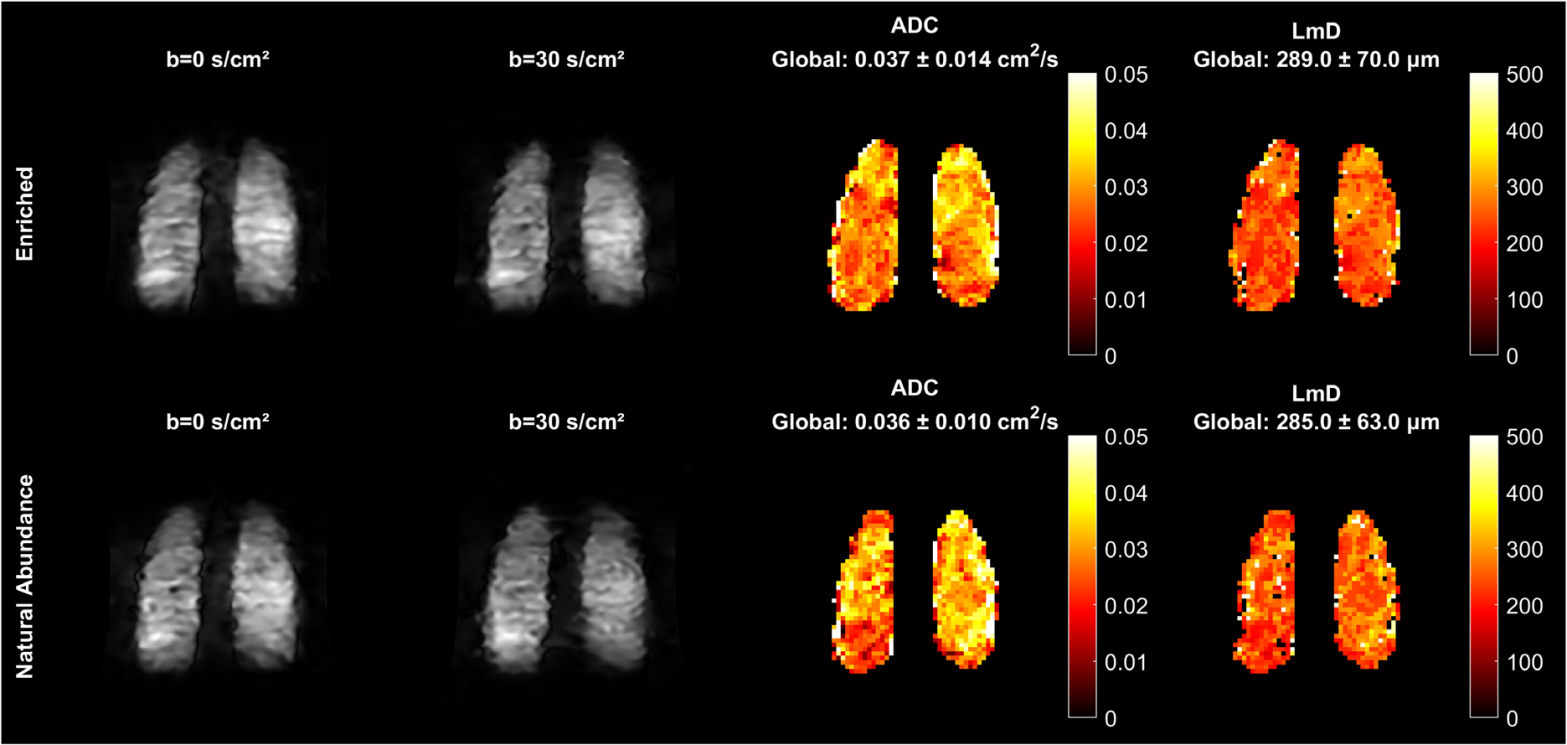
Prospective study. Enriched shows *b* = 0 s/cm^2^, *b* = 30 s/cm^2^ images, ADC and Lm_D_ maps from DL CS: DN + DR after inhalation of enriched xenon acquired at an acceleration factor of 5. Natural abundance shows *b* = 0 s/cm^2^, *b* = 30 s/cm^2^ images, ADC and Lm_D_ maps from DL CS: DN + DR with natural abundant xenon for the same volunteer. The global ADC and Lm_D_ values are indicated in the figure.

Figure 4 shows a comparison of DL CS versus DL CS: DN + DR for enriched xenon and natural abundant xenon for data acquired from one volunteer at an acceleration factor of 5. Similar to previous observations, the overall sharpness improved with DL CS: DN + DR, and the ADC and Lm_D_ maps exhibit preserved quantitative accuracy.

## 4 Discussion

In this work, we have demonstrated the feasibility and advantages of applying an extended deep learning and CS reconstruction pipeline (CS, denoising, and de‐ringing) to 3D ^129^Xe diffusion‐weighted MRI. The substantial SNR enhancement provided by DL reconstruction is particularly valuable for ^129^Xe diffusion‐weighted imaging, where the inherently lower SNR in high *b* value images often limits quantitative analysis.

The DL reconstruction approach differs from traditional denoising methods by integrating CS, denoising, and de‐ringing within a unified framework. This approach helps recover information and provide enhanced image quality which explains the superior performance, particularly at low SNR.

The retrospective study results showed visually improved image quality with a slight positive bias in ADC (5.4%) and slight negative bias in Lm_D_ (−0.78%), when compared with conventional CS reconstructions. The bias reduces to 1.46% for ADC and −0.3% for Lm_D_ when de‐ringing is removed. This aligns with previous findings by Stewart et al. [16] in ^129^Xe ventilation imaging with DL reconstruction, where ventilation metrics like ventilation defect percentage (VDP) and ventilation heterogeneity index (VHI) had a slightly higher bias with denoising and de‐ringing when compared to denoising only. Bland Altman analysis shows that the bias is within the statistical (95%) limits of agreement. This bias likely arises from the sharper structural boundaries due to the de‐ringing component of the DL reconstruction, which has been previously reported to increase sharpness at tissue interfaces [16]. Disabling de‐ringing improves the quantitative agreement with the gold‐standard but the reconstructed images lose the sharp boundaries generated due to de‐ringing (these being a major factor in improving visual image quality). The bias was consistent across subjects from healthy volunteers to patients with severe COPD. This suggests that DL reconstruction does not compromise the ability to detect focal pathological changes in lung microstructure.

During our analysis we also observed that the AIR Recon DL processing can produce subtle background artifacts, typically appearing as haze‐like patterns in high‐noise regions outside the lung region. These artifacts are minimal and do not affect quantitative analysis within the lung regions. This can be observed in the background in Figure 3.

The synthetic noise addition experiment provides valuable insights into the robustness of DL reconstruction across a range of SNR conditions. While the global ADC and Lm_D_ values look unaffected even after addition of noise, the MAE values increase with increasing noise levels which indicates divergence in the regional variations as noise level increases. However, visual inspection of the maps at three noise levels for the COPD dataset show that the main patterns of regional heterogeneity are preserved, thus demonstrating suitability for use of natural abundance xenon. The ability to maintain accurate diffusion metrics even at very low SNR levels is particularly important for clinical applications. These results suggest that DL reconstruction could potentially enable the use of natural abundance xenon instead of enriched xenon for diffusion‐weighted imaging while still obtaining reliable quantitative metrics. This was shown in the subsequent prospective study where healthy volunteers were scanned with natural abundance xenon and we observed good image quality and reliable quantitative metrics with DL reconstruction. Increasing the acceleration factor to 5 allowed a further reduction in the breath‐hold time from 17 to 14 s. This reduction is particularly important for patients with respiratory limitations who may struggle to maintain longer breath‐holds. Combined with the use of natural‐abundance xenon, these advances could significantly improve the economic and practical aspects of 3D ^129^Xe diffusion‐weighted MRI.

Some of the limitations of this work include the prospective experiments with natural abundance xenon and higher acceleration factors being performed on only healthy volunteers with normal lung function. Further studies in patients with pulmonary diseases are needed to assess the clinical utility of use of natural abundance xenon and DL reconstruction in a wider clinical setting. We have restricted our analysis to 3D Cartesian data acquired using SPGR at 1.5 T. Though the DL model can support both 2D and 3D scans, 3D SPGR is used routinely for diffusion imaging at our center. Also, the model used here cannot be applied directly to non‐Cartesian trajectories. Recently, there has been considerable interest in the use of non‐Cartesian trajectories for diffusion imaging [25, 26], and thus training and using a model for non‐Cartesian reconstruction will be the subject of future work. Finally, the specific DL reconstruction algorithm used in this study is currently only implemented on GE HealthCare scanners and alternative models that can support cross‐vendor studies will be important for the ^129^Xe MRI community.

## 5 Conclusion

DL‐based reconstruction significantly enhances SNR in ^129^Xe diffusion‐weighted MRI while preserving quantitative metrics (ADC and Lm_D_). This approach shows promise for the use of natural‐abundance xenon gas instead of more costly 129‐enriched xenon and may allow higher acceleration factors than conventional methods.

Our retrospective and prospective results extend the findings of Stewart et al. [16] for ventilation imaging to diffusion‐weighted imaging, where preserving quantitative accuracy is particularly critical for morphometric assessment of alveolar microstructure. In conclusion, the integration of DL‐based reconstruction for ^129^Xe diffusion‐weighted MRI shows promise for significantly enhancing the economic viability and clinical practicality of this technique.

## Conflicts of Interest

Rajagopalan Sundaresan, Sudhanya Chatterjee, Ramesh Venkatesan, and Jan Wolber are employees of GE HealthCare. The other authors declare no conflicts of interest.

## Supporting information


**Figure S1:** Deep learning‐based reconstruction pipeline for hyperpolarized ^129^Xe diffusion weighted MR. CS = compressed sensing reconstruction; DDC = distributed diffusion coefficient; SNR = signal‐to‐noise‐ratio.
**Figure S2:** (Prospective study) SNR comparison between deep learning CS reconstruction (DL CS) and deep learning CS reconstruction with denoising and de‐ringing (DL CS: DN + DR) after inhalation of enriched xenon and natural abundance xenon. Quoted SNR is the mean apparent SNR across all slices. The ADC and Lm_D_ maps from deep learning CS reconstruction with denoising and de‐ringing (DL CS: DN + DR) is also shown. Global ADC and Lm_D_ values are indicated in the figure.
**Figure S3:** (Retrospective and prospective study) Compressed sensing masks used for acceleration factors of 4 and 5. The *x*‐axis corresponds to *kz* dimension while the *y*‐axis corresponds to *ky* dimension. White points denote the sampled locations while the blue region denotes unsampled points. Note that the same masks are used across all *b* values.
**Figure S4:** (Retrospective study) Evaluation of four different denoising levels of 1, 0.75, 0.5, and 0.25 on a COPD dataset at *b* = 12 s/cm^2^ and *b* = 30 s/cm^2^. When denoising levels reduce, we can see more noise in the image background especially in *b* = 30 s/cm^2^ images. The increased sharpness in all the images is attributed to de‐ringing. While adjusting the denoising levels showed qualitative changes mostly in the background, there was no considerable quantitative changes observed. In all our experiments, we used 1.0 as denoising and de‐ringing levels due to low SNR characteristics of ^129^Xe imaging.
