# Supplementary material for: Deep Learning Reconstruction for 129Xe Diffusion‐Weighted MRI Enables Use of Natural Abundant Xenon and Improved Image Acceleration
Source: Magn Reson Med. 2025 Nov 20;95(4):2214–22. doi: 10.1002/mrm.70194 (PMC12850583; doi:10.1002/mrm.70194)
Supplement: Supplementary file 1 — Figure S1: Deep learning‐based reconstruction pipeline for hyperpolarized 129Xe diffusion weighted MR. CS = compressed sensing reconstruction; DDC = distributed diffusion coefficient; SNR = signal‐to‐noise‐ratio. Figure S2: (Prospective study) SNR comparison between deep learning CS reconstruction (DL CS) and deep learning CS reconstruction with denoising and de‐ringing (DL CS: DN + DR) after inhalation of enriched xenon and natural abundance xenon. Quoted SNR is the mean apparent SNR across all slices. The ADC and LmD maps from deep learning CS reconstruction with denoising and de‐ringing (DL CS: DN + DR) is also shown. Global ADC and LmD values are indicated in the figure. Figure S3: (Retrospective and prospective study) Compressed sensing masks used for acceleration factors of 4 and 5. The x‐axis corresponds to kz dimension while the y‐axis corresponds to ky dimension. White points denote the sampled locations while the blue region denotes unsampled points. Note that the same masks are used across all b values. Figure S4: (Retrospective study) Evaluation of four different denoising levels of 1, 0.75, 0.5, and 0.25 on a COPD dataset at b = 12 s/cm2 and b = 30 s/cm2. When denoising levels reduce, we can see more noise in the image background especially in b = 30 s/cm2 images. The increased sharpness in all the images is attributed to de‐ringing. While adjusting the denoising levels showed qualitative changes mostly in the background, there was no considerable quantitative changes observed. In all our experiments, we used 1.0 as denoising and de‐ringing levels due to low SNR characteristics of 129Xe imaging. [file MRM-95-2214-s001.docx]

**Supplementary figures**

**STAGE 1: Compressed sensing reconstruction**

**Undersampled**

**k-space**

64 x 52 x 18 matrix, 4x acceleration, b = 0, 12, 20, 30 cm2/s

**DL CS**

Deep learning-based regularization

**Reconstructed images**

4D dataset (3D spatial x-y-z + b value)

**STAGE 2: Image enhancement**

**Denoising and de-ringing**

Deep learning-based noise removal and ringing artifact removal

**Enhanced images**

Improved SNR with preserved quantitative metrics

**STAGE 3: Quantitative analysis**

**Stretched Exponential Model Analysis**

$\frac{\boldsymbol{S}_{\boldsymbol{b}}}{\boldsymbol{S}_{\boldsymbol{0}}}\boldsymbol{=}\boldsymbol{e}^{\boldsymbol{-(b * DD}\boldsymbol{C}^{\boldsymbol{\alpha}}\boldsymbol{)}}$***→ Mean Diffusive Length Scale (Lm_D_)***

Figure S1 Deep learning-based reconstruction pipeline for hyperpolarized ^129^Xe diffusion weighted MR.
CS = compressed sensing reconstruction; SNR = signal-to-noise-ratio; DDC = distributed diffusion coefficient


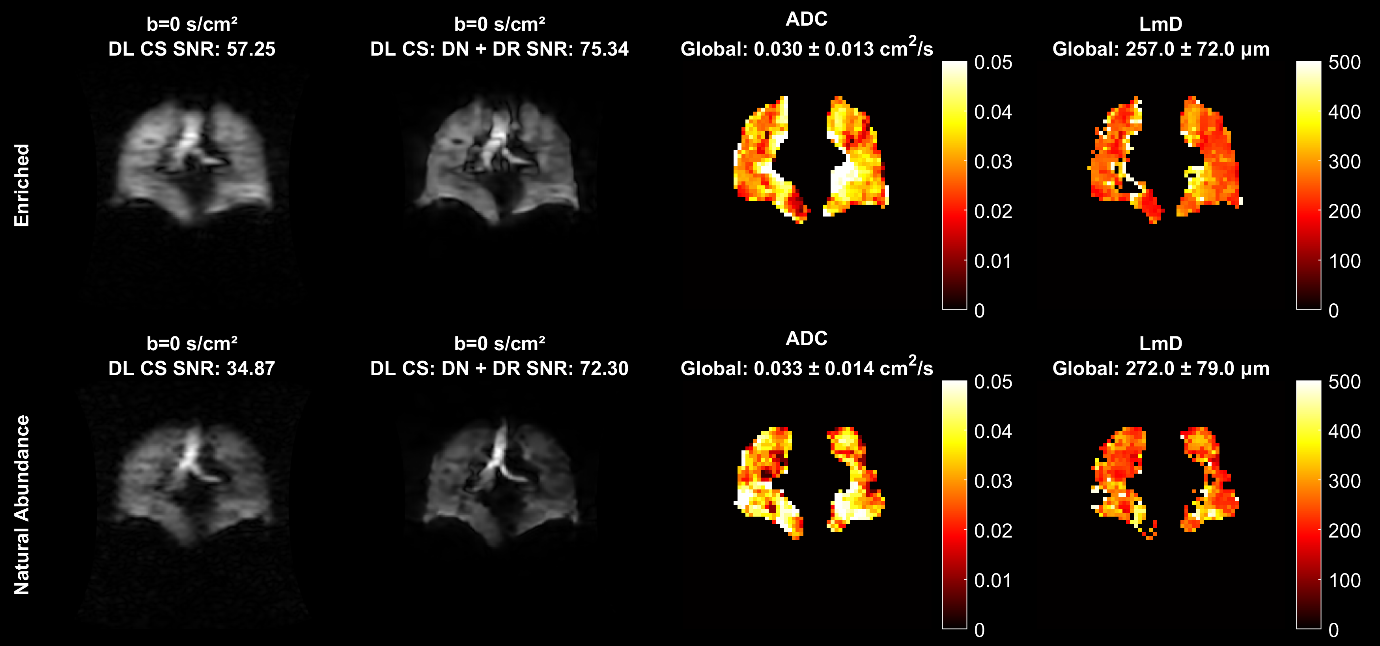


Figure S2 (Prospective study) SNR comparison between deep learning CS reconstruction (DL CS) and deep learning CS reconstruction with denoising and de-ringing (DL CS: DN + DR) after inhalation of enriched xenon and natural abundance xenon. Quoted SNR is the mean apparent SNR across all slices. The ADC and Lm_D_ maps from deep learning CS reconstruction with denoising and de-ringing (DL CS: DN + DR) is also shown. Global ADC and Lm_D_ values are indicated in the figure.


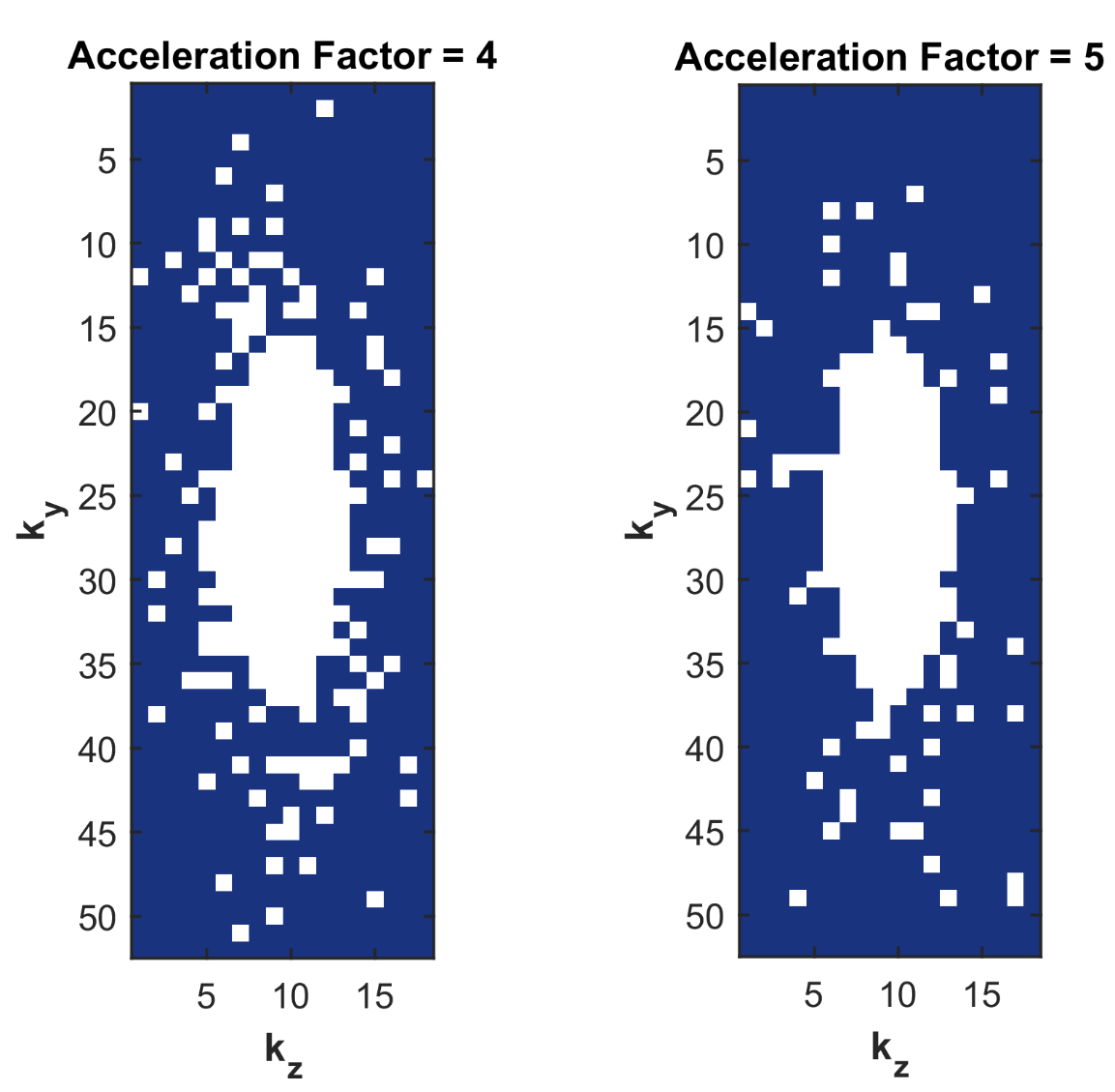


Figure S3 (Retrospective and prospective study) Compressed sensing masks used for acceleration factors of 4 and 5. The x-axis corresponds to kz dimension while the y-axis corresponds to ky dimension. White points denote the sampled locations while the blue region denotes unsampled points. Note that the same masks are used across all b-values.


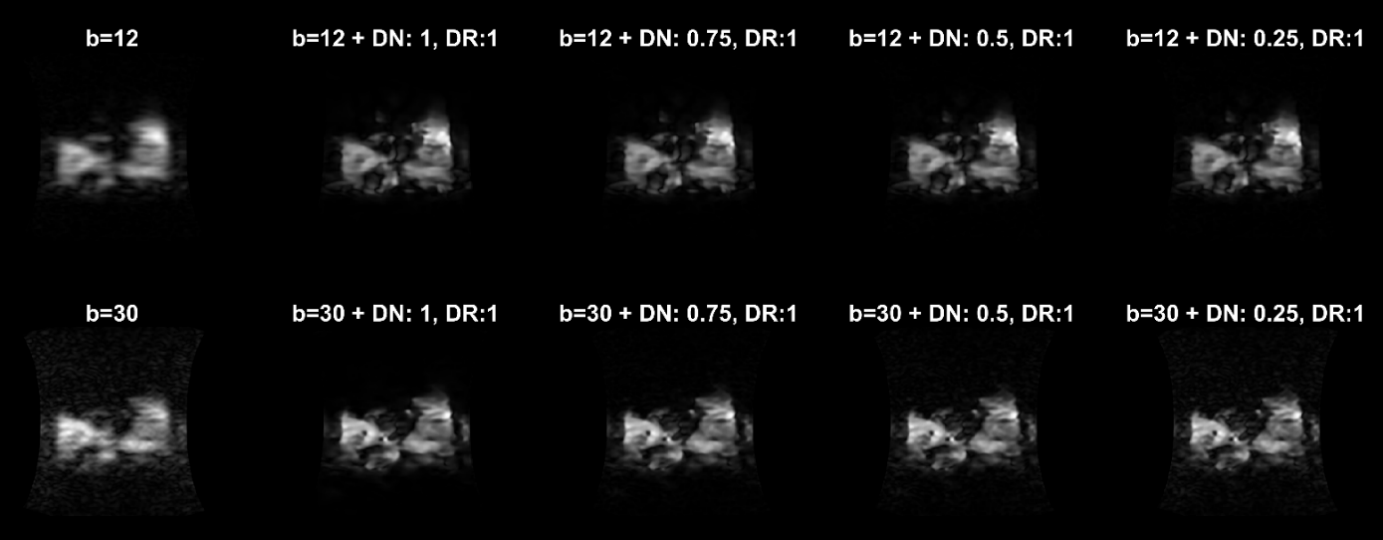


Figure S4 (Retrospective study) Evaluation of 4 different denoising levels of 1, 0.75, 0.5 and 0.25 on a COPD dataset at b=12 s/cm^2^ and b=30 s/cm^2^. When denoising levels reduce, we can see more noise in the image background especially in b=30 s/cm^2^ images. The increased sharpness in all the images is attributed to de-ringing. While adjusting the denoising levels showed qualitative changes mostly in the background, there was no considerable quantitative changes observed. In all our experiments, we used 1.0 as denoising and de-ringing levels due to low SNR characteristics of ^129^Xe imaging.
